# Reduced risk of shunt revision with adjustable valves: a population-based cohort study over three decades

**DOI:** 10.1007/s00701-026-06786-7

**Published:** 2026-02-03

**Authors:** Siiri Oksa, Roosa Kasurinen, Anssi Lipponen, Ville Leinonen, Antti J. Luikku

**Affiliations:** 1 Department of Neurosurgery, Institute of Clinical Medicine, University of Eastern Finland, Kuopio, 70211 Finland; 2 Department of Neurosurgery, Kuopio University Hospital, Kuopio, 70210 Finland; 3 Institute of Biomedicine, University of Eastern Finland, Kuopio, 70211 Finland

**Keywords:** Idiopathic normal pressure hydrocephalus, Revision, Prognosis

## Abstract

**Background:**

Idiopathic normal pressure hydrocephalus (iNPH) is a neurological disease characterized by ventriculomegaly and Hakim’s triad. At present, symptoms can be alleviated only by cerebrospinal fluid (CSF) shunt surgery. Yet, various complications after shunting may occur, occasionally requiring repeated shunt revisions. In this retrospective, population-based study, our objective was to compare revision rates and causes for revision surgeries between adjustable shunt valve and fixed-pressure valves in iNPH patients.

**Methods:**

Altogether 1220 patients were evaluated for possible iNPH at Kuopio University Hospital between 1991 and 2023. Probable iNPH was diagnosed in 809 patients who received their first ventriculoperitoneal shunt (VPS). Of the patient cohort, 566 were shunted using an adjustable valve (2008–2023) and 243 received a fixed pressure valve (1991–2012). Hospital records and nationwide registries were used to construct a timeline for each patient from the shunt insertion and until death (*n* = 430) or the end of 2023.

**Results:**

Overall revision rate was lower in iNPH patients receiving an adjustable valve (14% vs. 30%, *p* < .001, 95% CI 0.27—0.56). The incidence of multiple revisions was also lower in the adjustable valve group (27% vs. 32% *p* = 0.002, 95% CI 0.21—0.71). The most common cause for revision was peritoneal catheter malposition in the adjustable valve group (44%) and shunt underdrainage in the fixed-pressure valve group (25%).

**Conclusions:**

Adjustable shunt valves have decreased the need for shunt revision surgeries due to under- and overdrainage.

## Introduction

Idiopathic normal pressure hydrocephalus (iNPH) is a neurological disease, in which patients experience gait disturbance, urinary incontinence, and degradation of cognitive functions combined with ventriculomegaly [6]. Number of neurodegenerative disorders present with similar symptoms [14, 22], therefore iNPH remains underdiagnosed and undertreated [26]. A recent study has shown that the prevalence of iNPH is up to 3.7% amongst the population over 65 years of age [1].

CSF shunt surgery is currently the only recommendable and effective treatment for patients with iNPH [15, 16]. Depending on the patient and culture related issues, either a ventriculoperitoneal (VP), ventriculoatrial (VA), or lumboperitoneal (LP) shunt can be placed [11, 20]. The type of the valve utilized in the shunt system can be of a fixed or adjustable opening pressure setting. The functional mechanisms in fixed pressure valves are typically simpler than in the adjustable valves and therefore the price is also lower. The difficulty of fixed pressure valves comes into question in shunt over- or underdrainage cases. When the non-optimal opening pressure is causing symptoms in the patient, the valve must be replaced in a revision surgery. Using adjustable valves in shunt surgeries could therefore reduce the revision rate and compensate for the higher cost of these valves [7]. Adjusting the opening pressure of the valve could improve the outcome in patients with iNPH [24, 27], however there seems to be no difference in improvement after shunt surgery in iNPH patients comparing adjustable and fixed pressure valves [4].

Despite the initial response to shunt treatment, various causes might compromise the prognosis. Over- or underdrainage or obstruction of the shunt can hamper the response to treatment [2]. Shunt infections have shown to occur rarely [5, 9], but can be severe. In VP-shunts, most of these complications seem to occur in the first year after shunting [19]. However, well-timed shunt revision surgery has resulted in clinical improvement in most shunt malfunction -cases [8, 21].

Since no other effective treatment for iNPH exists so far, malfunction cases should be detected rapidly. Recognizing shunt malfunction is based on clinical suspicion due to patient’s returning symptoms, signs of infection or other change in condition. As in primary iNPH diagnostics, CSF tap testing can also be used to predict the outcome of revision surgery and therefore avoid needless shunt revisions [18].

A recent study showed that the use of adjustable shunt valves might result in better revision-free survival in iNPH patients [3, 23]. In our population-based study cohort, 809 patients were diagnosed with iNPH and received their first VPS at Kuopio University Hospital (KUH) over three decades between 1991 and 2023. The focus of the study was the 566 iNPH patients with an adjustable shunt valve (2008–2023) compared to the 243 patients with a fixed pressure valve (1991–2012). The objective was to compare the revision rates and analyze the causes for revision surgery in these two groups during the follow-up until death (*n* = 430) or December 2023. Our hypothesis was that the revision rates, especially considering shunt over- and underdrainage have lowered as the use of adjustable shunt valves began.

## Methods

### Study population

**Fig. 1:**
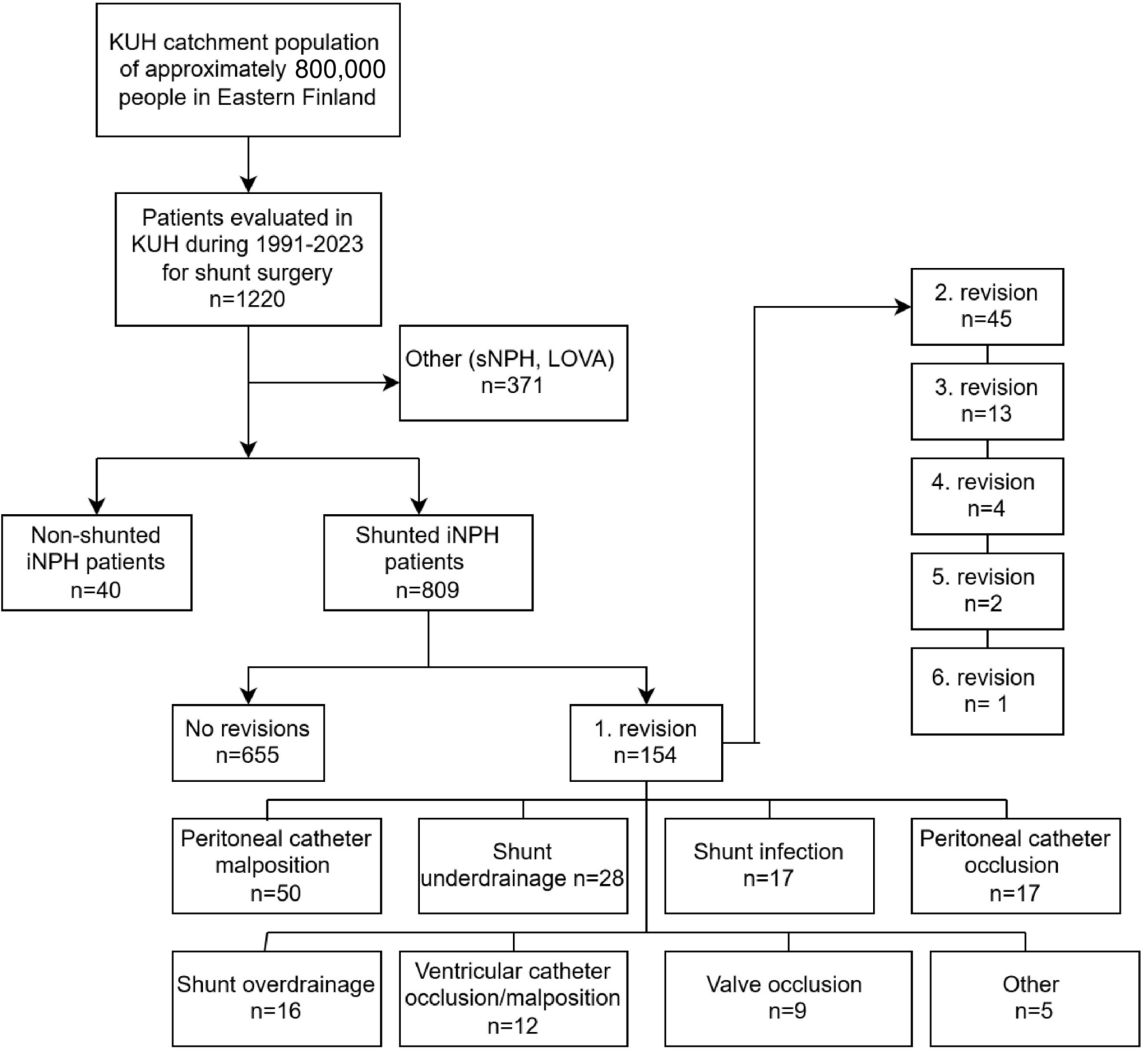
The study cohort of shunted idiopathic normal pressure hydrocephalus (iNPH) patients from Kuopio University Hospital catchment population in 1991–2023 presented as a flow chart. Causes for first shunt revision are subcategorized based on patients’ clinical symptoms, results of possible cerebrospinal fluid (CSF) tap tests, infusion tests, surgical findings and bacteriological data. Abbreviations: KUH = Kuopio University Hospital, sNPH = secondary normal pressure hydrocephalus, LOVA = longstanding overt vetriculomegaly in adults

KUH catchment population consists of approximately 800,000 people in Eastern Finland. Of this population, 1220 consecutive adult patients were evaluated due to either secondary normal pressure hydrocephalus (sNPH), long-standing overt ventriculomegaly in adults (LOVA), or iNPH in between 1991 and 2023. Our final study cohort consists of 809 iNPH patients diagnosed and treated with a CSF shunt according to protocol [10] presented in Fig. 1.

### Clinical data retrieval sources

Each patient’s medical records during follow-up were analyzed for shunt valve types and shunt revisions. The clinical data used in the study was retrieved from the Kuopio NPH registry, KUH neurosurgical operative reports on shunt installations and revisions, follow-up reports, and referrals from Central Hospitals in KUH catchment area. The data was collected and processed for each patient from shunt insertion until death (*n* = 430) or the end of the year 2023. Malfunctions were subcategorized according to the probable cause for malfunction. Different causes were detected by typical clinical symptoms, results of possible CSF tap tests, infusion tests, surgical findings, and bacteriological data. The characteristics of the patients are described in Table 1.

**Table 1:** Study population of 809 iNPH patients treated with a ventriculoperitoneal shunt between 1991 and 2023

	Adjustable valve (2008–2023) *n* = 566	Fixed pressure valve (1991–2012) *n* = 243	*p*	95% CI
Females	259 (46%)	135 (56%)	0.073	1.12—2.05
Median age at primary shunting, years	75 (70–79)	72 (68–77)	0.115	
First revision	81 (14%)	73 (30%)	** < 0.001**	0.27—0.56
Median time to first revision, weeks	18 (4–84)	17 (8–120)	0.472	
Revision cause				
Peritoneal catheter malposition	35 (6,2%)	15 (6,2%)	0.995	0.54—1.87
Median time to revision, weeks	9 (0–111)	67 (1–525)		
Peritoneal catheter occlusion	10 (1,7%)	7 (2,9%)	0.271	0.20—1.48
Median time to revision, weeks	61 (16–200)	83 (3–373)	** < 0.001**	0.10—0.50
Shunt underdrainage	10 (1,7%)	18 (7,4%)		
Median time to revision, weeks	186 (59–565)	151 (5–751)		
Shunt infection	11 (1,9%)	6 (2,5%)	0.602	0.29—2.14
Median time to revision, weeks	6 (0–103)	24 (0–120)		
Shunt overdrainage	4 (0,7%)	12 (4,9%)	** < 0.001**	0.05—0.48
Median time to revision, weeks	186 (13–222)	100 (2–398)		
Ventricular catheter occlusion/malposition	6 (1,1%)	6 (2,5%)	0.321	0.15—1.69
Median time to revision, weeks	8 (0–35)	15 (0–46)		
Valve occlusion	3 (0,5%)	6 (2,5%)	0.057	0.06—1.07
Median time to revision, weeks	127 (112–182)	87 (9–568)		
Other^*^	2 (0,4%)	3 (1,2%)	0.901	0.08—9.51
Median time to revision, weeks	134 (18–250)	396 (31–568)		
Two or more revisions per patient	22 (3,9%)	23 (9,5%)	**0.002**	0.21—0.71
Deaths during follow-up	218 (39%)	212 (87%)	** < 0.001**	0.06—0.13

Variables are shown in median (IRS) or number (percent)

Fisher exact test was used for categorical variables, Mann–Whitney *U*-test for continuous

^*^Other causes for shunt revision include catheter disconnection, surgery complication and trauma

### Shunt surgery and follow-up

All shunt installations and revisions in the KUH catchment area have been conducted by KUH Neurosurgery. The follow-up of shunted patients has also been arranged by KUH in Neurosurgery and Neurology Departments of four Central Hospitals in KUH area.

### Types of shunt valves in iNPH patients

During the study period from 1991 to 2023, shunt valves with two different pressure mechanisms were used in the treatment of iNPH patients.

1. Fixed pressure valves (*n* = 243).

Exclusively from 1991 until 2007 (*n *=207) and occasionally from 2008 until 2012 (*n*=36)

2. Adjustable valves (*n* = 566).

Used since 2008 and became the main valve type in 2009.

### Statistical analysis

Statistical analysis was performed with SPSS version 29.0 (SPSS, Inc., Chicago, IL). The differences were considered statistically significant if the *p*-value was < 0.05. Categorical variables were evaluated by x[2] test. Differences in shunt survival concerning the first shunt revision surgery between adjustable and fixed-pressure shunt valves were analyzed with Kaplan–Meier analysis. Independent risk factors for the first revision were identified using Cox regression analysis.

## Results

**Fig. 2:**
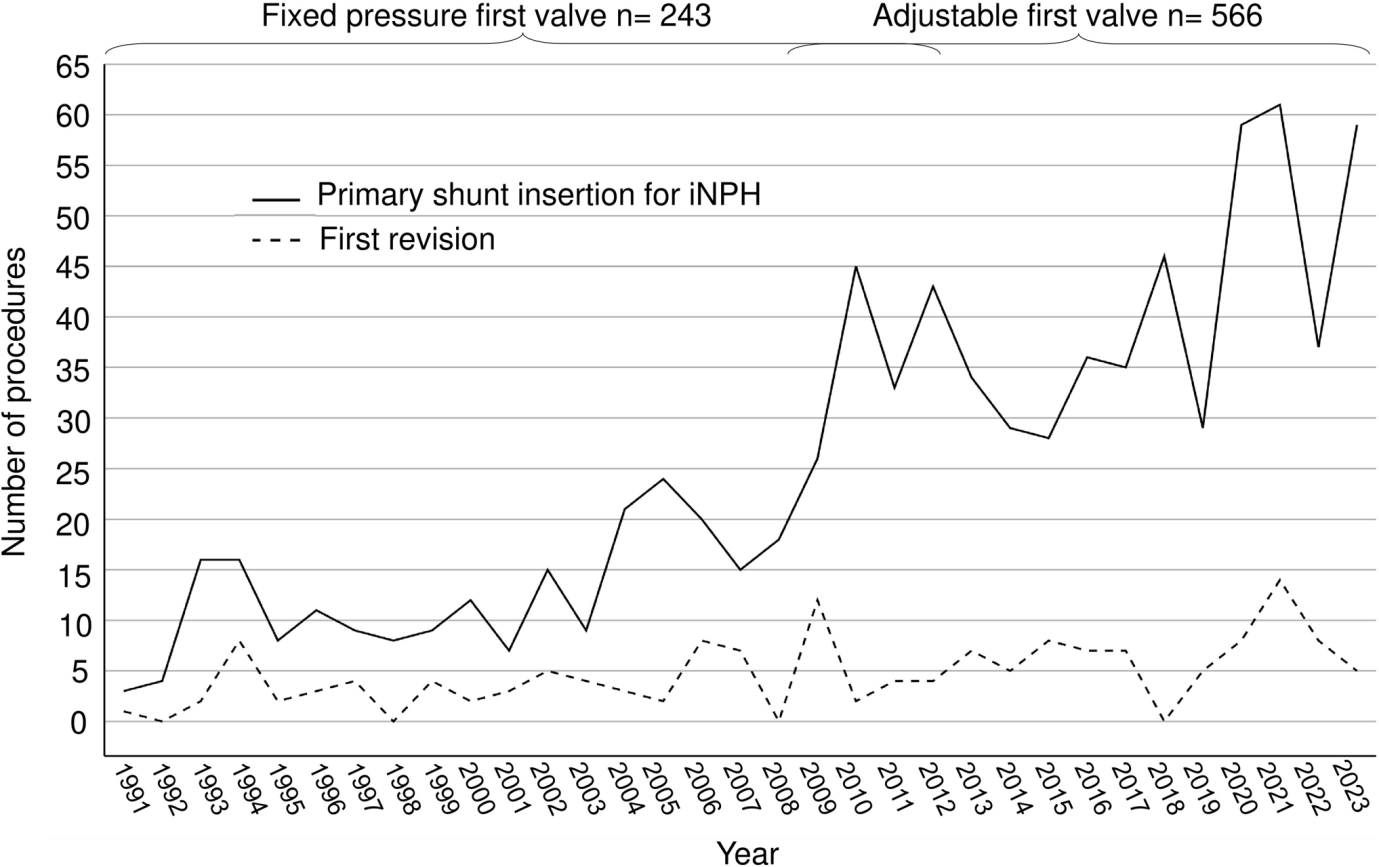
Timelines of two shunt pressure mechanisms in 809 iNPH patients. Altogether 1220 consecutive patients were evaluated for iNPH between 1991 and 2023 at Kuopio University Hospital (KUH) from its Eastern Finnish catchment population. iNPH was diagnosed and treated by ventriculoperitoneal shunting (VPS) in 809 patients. The annual numbers of primary shunt insertions for iNPH (*n* = 809) and first shunt revisions (*n* = 154) are presented in this figure. From 1991 to 2007 fixed pressure shunt valves were primarily inserted. The insertion of adjustable shunt valves began in 2008 and became the main valve type in 2009. After 2012 only adjustable valves have been inserted in iNPH patients

From the final study population of 809 iNPH patients, 154 (19%) underwent the first shunt revision in a median of 18 weeks (IQR 4–88). Median follow-up time was 4.9 years until death (*n* = 430) or December 2023, in a total of 5072 follow-up years. Annual revision rates in iNPH patients are presented in Fig. 2.

### Adjustable valves (*n* = 566; 2008–2023)

Of the 809 shunted iNPH patients, 566 (70%) had an adjustable shunt valve (Table 1). The median follow-up time from shunt insertion, until death (*n* = 218) or the end of the year 2023, was 3.9 years (IQR 1.9—6.7).

Of the 566 patients, 485 (86%) patients survived without a revision surgery for a median of 3.8 years, whereas shunt revision was needed in 81 (14%) patients. The median time to revision from shunt insertion was 18 weeks from shunt insertion. Two or more revisions were required in 22 (27%) of the 81 revised patients.

Peritoneal catheter malposition (*n* = 36) was the most common cause for shunt revision in a median time of 9 weeks to revision from shunt insertion. The second most common cause for revision was infection (*n* = 11). The median time to revision in these cases was 6 weeks. The most common findings in bacteriological cultures were Staphylococcus epidermidis (*n* = 3) and Staphylococcus aureus (*n* = 2). In three cases the bacteriological cultures remained negative, despite firm clinical suspicion of infection.

### Different valve types in adjustable valves

The most frequently used adjustable shunt valves were Medtronic Strata II (*n* = 276, 48.8%) and Strata (*n* = 199, 35.2%). Less commonly used valve types included Miethke M.Blue Plus (*n* = 36), Codman Certas Plus with SiphonGuard (*n* = 31), Miethke proGAV 2.0 (*n* = 18), Miethke proGAV (*n* = 4) and Medtronic Strata NSC (*n* = 2).

All adjustable valves used in our study cohort were pressure-controlled. We grouped the 566 iNPH patients with an adjustable valve in two groups depending whether their shunt valve included a gravitational unit (Miethke M.Blue Plus, Miethke proGAV 2.0, Miethke proGAV, *n* = 58) or no gravitational unit (Medtronic Strata II, Strata, Codman Certas plus with SiphonGuard, Medtronic Strata NSC, *n* = 508).

Of the 58 patients with a gravitational unit, 50 (86%) patients survived without a revision surgery for a median of 2.3 years, whereas 8 (14%) patients underwent revision surgery. The median time to revision from shunt insertion was 64 weeks from shunt insertion. In the non-gravitational valve group, 435 (86%) patients survived without revision surgery for a median of 4.0 years. Shunt revision was needed in 81 (14%) patients in a median time of 18 weeks from shunt insertion.

In the analysis, no statistically significant difference in the risk of first shunt revision was observed between adjustable valves with and without a gravitational unit.

### Fixed pressure valves (*n* = 243; 1991–2012)

Of the 809 shunted patients, 243 (30%) had a fixed pressure valve (Table 1). The median follow-up time from shunt insertion, until death (*n* = 212) or end of the year 2023 was 9.0 years (IQR 4.8–14.2).

Of the 243 patients, 170 (70%) survived without a revision surgery for a median of 9.2 years, whereas shunt revision was needed in 73 (30%) patients. The median time to revision was 17 weeks from shunt insertion. Multiple revisions were needed in 23 (32%) of the 73 revised patients.

Shunt underdrainage (*n* = 18) was the most common cause for revision in a median time of 151 weeks to revision from shunt insertion (Table 1). The second most common cause for revision was peritoneal catheter malposition (*n* = 15). The median time to revision in these cases was 67 weeks.

### Risk of revision with fixed pressure valves and adjustable valves

**Fig. 3:**
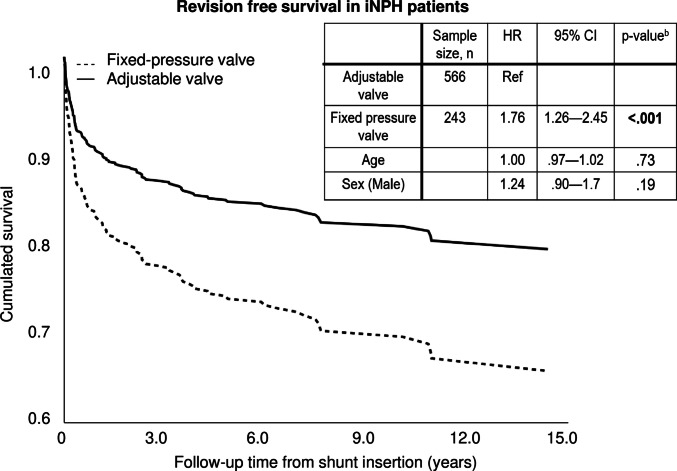
Revision-free survival in 809 iNPH shunted patients with two different shunt pressure mechanisms using Cox regression in 1991–2023. Abbreviations: HR = hazard ratio, CI = confidence interval, Ref = reference

Over the entire follow-up, the risk for the first revision was higher for the patients with fixed-pressure valve (HR = 1.76; *p* < 0.001, 95% CI 1.26—2.45; Fig. 3). Sex and age at shunt insertion did not show significance in the analysis.

## Discussion

In our population-based study cohort, iNPH was diagnosed and treated by ventriculoperitoneal shunting in 809 patients using fixed pressure (1991–2012) or adjustable shunt valves (2008–2023). During the follow-up, the rate of first revision surgery was 19%.

In historic fixed pressure valves, the revision rate was 30% in a median time of 17 weeks to revision. As hypothesized, shunt failures due to hydrodynamic causes—shunt under- and overdrainage—were dominant in fixed pressure valves as the setting cannot be modified. Shunt underdrainage was the most common cause (7.4%) and shunt overdrainage was the third most common cause (4.9%) for revision surgery. The second most common cause for revision surgery in fixed-pressure valves was malposition of the peritoneal catheter (6.2%).

The revision rate in the adjustable valve group was 14%, being significantly lower compared to the fixed pressure valves (*p* < 0.001, 95% CI 0.27—0.56). The most common causes for revision surgery were peritoneal catheter malposition (6.2%) and shunt infection (1.9%). As hypothesized, hydrodynamic shunt failures were significantly less common in adjustable shunt valves (*p* < 0.001, 95% CI 0.10 —0.50 in underdrainage and 95% CI 0.05—0.48 in overdrainage), although shunt underdrainage was the third most common cause for revision (1.7%). The use of adjustable shunt valve also resulted in a better revision-free survival (*p* < 0.001, 95% CI 1.26—2.45).

The tax-paid Finnish healthcare system brings significant strengths to this study. The five Finnish University hospitals have their own catchment areas from which all patients receive care unselected. All patients’ contacts to healthcare are archived in national registries and can be inspected by professionals using the Finnish identity codes. Causes of death are also documented in national registries. This offers long-term follow-up with almost no patients lost to follow-up.

However, there are also limitations. Although national registries allow comprehensive data collection, the nature of the study is retrospective. In our study, the follow-up time for patients with adjustable shunt valves is shorter than for patients with fixed-pressure shunt valves as the insertion of adjustable shunt valves began only in 2008. Thus, the later shunt failures in the adjustable valve group may be underrepresented regardless of the adjusted statistical models used in the analysis.

As the follow-up period in this study spans over multiple decades, changes in surgical practice, diagnostics, and perioperative care also likely contribute to improved survival beyond the independent effect of the valve type. For peritoneal catheter malposition, median time to revision was 67 weeks in the fixed-pressure valve group versus 9 weeks in the adjustable valve group. This is possibly also reflecting earlier detection of shunt malfunction due to improved postoperative imaging rather than the effect of the valve type.

Although all patients with suspected shunt malfunction in the KUH catchment area are referred to KUH Neurosurgery, some cases may be misinterpreted as age-related frailty or other conditions and therefore are not referred. However, this limitation should occur similarly across decades and regardless of the shunt valve type and is thus unlikely to affect the results of this study.

Even if the shunt surgery is complication-free and the patient does not suffer any shunt malfunctions, accompanying disorders still might compromise the survival of the patient. INPH patients are at significant risk of developing dementia or cognitive impairment related to iNPH itself [12] or some comorbidities such as Alzheimer’s disease (AD) [17], or vascular dementia (VD). Less frequently iNPH is accompanied by Parkinson's disease (PD), dementia with Lewy bodies (DLB), frontotemporal dementia (FTD), or corticobasal degeneration (CBD) [13, 25]. Such comorbidities were also present in our cohort and may complicate symptom reporting and clinical decision-making regarding revision surgery and therefore influence our results. The impact of comorbid dementia in the survival of iNPH patients is an important issue for further study.

Follow-up is required for iNPH patients to detect possible complications and comorbidities rapidly. Clinical outcomes considering gait improvement, cognitive performance and quality of life are also important factors in the prognosis of iNPH patients. These variables were not systematically assessed in this study and therefore require further research.

## Conclusions

The use of adjustable shunt valves in iNPH patients has significantly decreased the rates of shunt revisions, especially considering hydrodynamic shunt failures. In this study population, the use of adjustable shunt valves also resulted in a better revision-free survival. This suggests that the use of adjustable shunt valves is recommendable in iNPH patients despite the higher cost compared to fixed-pressure valves.

## Data Availability

Data sets supporting the findings of this study are available anonymized from the corresponding author on reasonable request. Data sets are located in controlled access data storage in Kuopio University Hospital.

## References

[1] Andersson J, Rosell M, Kockum K, Lilja-Lund O, Söderström L, Laurell K (2019) Prevalence of idiopathic normal pressure hydrocephalus: a prospective, population-based study. PLoS ONE 14(5):e0217705. 10.1371/journal.pone.0217705 31141553 10.1371/journal.pone.0217705 PMC6541279

[2] Bettag C, von der Brelie C, Freimann FB, Thomale UW, Rohde V, Fiss I (2022) In vitro testing of explanted shunt valves in hydrocephalic patients with suspected valve malfunction. Neurosurg Rev 45(1):571–583. 10.1007/s10143-021-01564-8 34027574 10.1007/s10143-021-01564-8 PMC8827297

[3] Chen KH, Hsu PW, Wu BC et al (2023) Long-term follow-up and comparison of programmable and non-programmable ventricular cerebrospinal fluid shunts among adult patients with different hydrocephalus etiologies: a retrospective cohort study. Acta Neurochir (Wien) 165(9):2551–2560. 10.1007/s00701-023-05734-z 37553445 10.1007/s00701-023-05734-z PMC10477099

[4] Farahmand D, Sæhle T, Eide PK, Tisell M, Hellström P, Wikkelsö C (2016) A double-blind randomized trial on the clinical effect of different shunt valve settings in idiopathic normal pressure hydrocephalus. J Neurosurg 124(2):359–367. 10.3171/2015.1.JNS141301 26315004 10.3171/2015.1.JNS141301

[5] Feletti A, d’Avella D, Wikkelsø C et al (2019) Ventriculoperitoneal shunt complications in the European idiopathic normal pressure hydrocephalus multicenter study. Oper Neurosurg 17(1):97–102. 10.1093/ons/opy232 30169650 10.1093/ons/opy232

[6] Gavrilov GV, Gaydar BV, Svistov DV et al (2019) Idiopathic normal pressure hydrocephalus (Hakim-Adams Syndrome): clinical symptoms, diagnosis and treatment. Psychiatr Danub 31(Suppl 5):737–744 32160166

[7] Giordan E, Palandri G, Lanzino G, Murad MH, Elder BD (2018) Outcomes and complications of different surgical treatments for idiopathic normal pressure hydrocephalus: a systematic review and meta-analysis. J Neurosurg: 1–13. 10.3171/2018.5.JNS1875. (Published online November 1) 10.3171/2018.5.JNS1875 30497150

[8] Grasso G, Torregrossa F, Leone L, Frisella A, Landi A (2019) Long-term efficacy of shunt therapy in idiopathic normal pressure hydrocephalus. World Neurosurg 129:e458–e463. 10.1016/j.wneu.2019.05.183 31154105 10.1016/j.wneu.2019.05.183

[9] Hung AL, Vivas-Buitrago T, Adam A et al (2017) Ventriculoatrial versus ventriculoperitoneal shunt complications in idiopathic normal pressure hydrocephalus. Clin Neurol Neurosurg 157:1–6. 10.1016/j.clineuro.2017.03.014 28347957 10.1016/j.clineuro.2017.03.014

[10] Junkkari A, Luikku AJ, Danner N et al (2019) The Kuopio idiopathic normal pressure hydrocephalus protocol: initial outcome of 175 patients. Fluids Barriers CNS 16(1):21. 10.1186/s12987-019-0142-9 31340831 10.1186/s12987-019-0142-9 PMC6657079

[11] Kazui H, Miyajima M, Mori E, Ishikawa M (2015) Lumboperitoneal shunt surgery for idiopathic normal pressure hydrocephalus (SINPHONI-2): an open-label randomised trial. Lancet Neurol 14(6):585–594. 10.1016/S1474-4422(15)00046-0 25934242 10.1016/S1474-4422(15)00046-0

[12] Koivisto AM, Alafuzoff I, Savolainen S et al (2013) Poor cognitive outcome in shunt-responsive idiopathic normal pressure hydrocephalus. Neurosurgery 72(1):1–8. 10.1227/NEU.0b013e31827414b3 23037817 10.1227/NEU.0b013e31827414b3

[13] Koivisto AM, Kurki MI, Alafuzoff I et al (2016) High risk of dementia in ventricular enlargement with normal pressure hydrocephalus related symptoms1. J Alzheimers Dis 52(2):497–507. 10.3233/JAD-150909 27031474 10.3233/JAD-150909

[14] Korhonen VE, Solje E, Suhonen NM et al (2017) Frontotemporal dementia as a comorbidity to idiopathic normal pressure hydrocephalus (iNPH): a short review of literature and an unusual case. Fluids Barriers CNS 14(1):10. 10.1186/s12987-017-0060-7 28420385 10.1186/s12987-017-0060-7 PMC5395836

[15] Luciano M, Holubkov R, Williams MA et al (2023) Placebo-controlled effectiveness of idiopathic normal pressure hydrocephalus shunting: a randomized pilot trial. Neurosurgery 92(3):481–489. 10.1227/neu.0000000000002225 36700738 10.1227/neu.0000000000002225 PMC9904195

[16] Luciano MG, Williams MA, Hamilton MG et al (2025) A randomized trial of shunting for idiopathic normal-pressure hydrocephalus. N Engl J Med. 10.1056/NEJMoa2503109 40960253 10.1056/NEJMoa2503109 PMC12682072

[17] Luikku AJ, Hall A, Nerg O et al (2019) Predicting development of Alzheimer’s disease in patients with shunted idiopathic normal pressure hydrocephalus. J Alzheimers Dis 71(4):1233–1243. 10.3233/JAD-190334 31498122 10.3233/JAD-190334

[18] Lundkvist B, Koskinen LOD, Birgander R, Eklund A, Malm J (2011) Cerebrospinal fluid dynamics and long-term survival of the Strata valve in idiopathic normal pressure hydrocephalus. Acta Neurol Scand 124(2):115–121. 10.1111/j.1600-0404.2010.01432.x 21039363 10.1111/j.1600-0404.2010.01432.x

[19] Merkler AE, Ch’ang J, Parker WE, Murthy SB, Kamel H (2017) The rate of complications after ventriculoperitoneal shunt surgery. World Neurosurg 98:654–658. 10.1016/j.wneu.2016.10.136 27826086 10.1016/j.wneu.2016.10.136 PMC5326595

[20] Nakajima M, Yamada S, Miyajima M et al (2021) Guidelines for management of idiopathic normal pressure hydrocephalus (Third Edition): endorsed by the Japanese Society of Normal Pressure Hydrocephalus. Neurol Med Chir (Tokyo) 61(2):63–97. 10.2176/nmc.st.2020-0292 33455998 10.2176/nmc.st.2020-0292 PMC7905302

[21] Pujari S, Kharkar S, Metellus P, Shuck J, Williams MA, Rigamonti D (2008) Normal pressure hydrocephalus: long-term outcome after shunt surgery. J Neurol Neurosurg Psychiatry 79(11):1282–1286. 10.1136/jnnp.2007.123620 18356257 10.1136/jnnp.2007.123620

[22] Reeves BC, Karimy JK, Kundishora AJ et al (2020) Glymphatic system impairment in Alzheimer’s disease and idiopathic normal pressure hydrocephalus. Trends Mol Med 26(3):285–295. 10.1016/j.molmed.2019.11.008 31959516 10.1016/j.molmed.2019.11.008 PMC7489754

[23] Rinaldo L, Bhargav AG, Nesvick CL, Lanzino G, Elder BD. Effect of fixed-setting versus programmable valve on incidence of shunt revision after ventricular shunting for idiopathic normal pressure hydrocephalus. J Neurosurg. Published online June 7, 2019:1–9. 10.3171/2019.3.JNS183077 10.3171/2019.3.JNS183077 31174190

[24] Sæhle T, Farahmand D, Eide PK, Tisell M, Wikkelsö C (2014) A randomized controlled dual-center trial on shunt complications in idiopathic normal-pressure hydrocephalus treated with gradually reduced or “fixed” pressure valve settings. J Neurosurg 121(5):1257–1263. 10.3171/2014.7.JNS14283 25192478 10.3171/2014.7.JNS14283

[25] Sakurai A, Tsunemi T, Ishiguro Y, Okuzumi A, Hatano T, Hattori N (2022) Comorbid alpha synucleinopathies in idiopathic normal pressure hydrocephalus. J Neurol 269(4):2022–2029. 10.1007/s00415-021-10778-1 34468800 10.1007/s00415-021-10778-1

[26] Williams MA, Nagel SJ, Luciano MG et al (2019) The clinical spectrum of hydrocephalus in adults: report of the first 517 patients of the Adult Hydrocephalus Clinical Research Network registry. J Neurosurg 132(6):1773–1784. 10.3171/2019.2.JNS183538 31125971 10.3171/2019.2.JNS183538

[27] Zemack G, Romner B (2002) Adjustable valves in normal-pressure hydrocephalus: a retrospective study of 218 patients. Neurosurgery 51(6):1392–1400 (discussion 1400-1402) 12445344

